# Supplementary material for: DNA as a quantum system in evolution
Source: PLoS One. 2026 Mar 20;21(3):e0344520. doi: 10.1371/journal.pone.0344520 (PMC13004412; doi:10.1371/journal.pone.0344520)
Supplement: S1 File — (DOCX) [file pone.0344520.s001.docx]

Supporting information for

# DNA as a quantum system in evolution

Nahuel Aquiles Garcia

Corresponding author: [info@gecorp.com.ar](mailto:info@gecorp.com.ar)

# Supplementary materials and methods

All the analyses were performed on the Mycobacterium tuberculosis genome and its annotations (https://[www.ncbi.nlm.nih.gov/datasets/genome/GCA_013010385.1/).](http://www.ncbi.nlm.nih.gov/datasets/genome/GCA_013010385.1/)) The developed codes are available at https://github.com/nahuelaquiles/-DNA-as-system-computer-to-measure-time

## Shannon Entropy Calculation

Shannon entropy (H(X)) was used to quantify the informational diversity of DNA sequences in coding and non-coding regions of the *Mycobacterium tuberculosis* genome. The calculation measures the uncertainty or randomness in the sequence based on the distribution of nucleotide frequencies.

The entropy is calculated using the formula:

$$H\left( X \right)=-\sum_{i=1}^{n} p\left( x_{i} \right)\log_{2}p\left( x_{i} \right)$$

Where:

- H(X) is the Shannon entropy of the sequence X.
- p(x_i_) is the probability (relative frequency) of the i-th nucleotide in the sequence.
- n is the number of unique nucleotides (e.g., A, T, C, G) in the sequence.
- The logarithm is in base 2 to express entropy in bits.

## Steps for Calculation:

1. **Segregation of Genome**: The *Mycobacterium tuberculosis* genome was divided into coding and non-coding regions based on the provided annotations (<https://www.ncbi.nlm.nih.gov/datasets/genome/GCA_013010385.1/>).
2. **Frequency Count**: For each region, the occurrences of each nucleotide (A,T,C,G) were counted.
3. **Probability Calculation**: These counts were normalized by dividing each nucleotide count by the total sequence length to obtain the probabilities (p(x_i_)).
4. **Entropy Contribution**: For each nucleotide, the contribution to entropy was calculated as: −p(x_i_)log_2_p(x_i_).
5. **Summation**: The entropy contributions of all nucleotides were summed to obtain the total entropy for the sequence.

This method was applied to the coding and non-coding regions separately, allowing for comparative analysis of informational complexity.

## Von Neumann Entropy Calculation

Von Neumann entropy (S(ρ)) is a crucial measure of quantum informational diversity, capturing the complexity and coherence of quantum states in coding and non-coding DNA regions. This measure provides insights into how these genomic regions manage quantum information, offering a comparative view of their informational density. The

code for the calculation is available at [https://github.com/aquilito/DNA-as-quantum-](https://github.com/aquilito/DNA-as-quantum-computer-to-measure-time) [computer-to-measure-time](https://github.com/aquilito/DNA-as-quantum-computer-to-measure-time).

## Mathematical Framework

1. **Density Matrix (ρ)**: The density matrix is a quantum mechanical representation of the system's state, capturing the statistical properties of the qubit states:

$$\rho=\frac{1}{n}\sum_{i=1}^{n} \left| \psi_{i}\rangle\langle\psi_{i} \right|$$

n is the number of nucleotides in the sequence.

∣ψ_i_⟩ is the quantum state of the i-th nucleotide, determined by the DNA-to-qubit mapping.

1. **Von Neumann Entropy (S(ρ))**: Once the density matrix is constructed, the entropy is calculated as:

$$S\left( \rho\right)=-\sum_{i} \lambda_{i}\log_{2}\lambda_{i}$$

Where λi are the eigenvalues of the density matrix. Eigenvalues represent the probabilities of different quantum states, and their logarithmic contribution measures uncertainty.

## DNA to Qubit Mapping

To translate DNA sequences into quantum states:

## Mapping Strategy:

- - **A (Adenine)**: [1, 0] (|0⟩)
  - **T (Thymine)**: [0, 1] (|1⟩)
  - **C (Cytosine)**: [1, 1] (superposition state |0⟩ + |1⟩)
  - **G (Guanine)**: [0, 0] (collapsed state)

These mappings embed the nucleotide alphabet into a two-level subspace plus an erasure/vacuum state. The full sequence state (or the resulting density matrix) is normalized so that total probability (or trace) equals 1.

Note on generality: The encoding is applied site-by-site, so it is defined for any DNA sequence s = s1...sN of arbitrary length and composition. The "four cases" correspond to the four nucleotide symbols, not a restriction on sequence generality.

Note on the guanine state. The vector [0, 0] is not a normalized qubit; it is used as a shorthand for an erasure/vacuum state |vac> orthogonal to {|0>, |1>}. In density-matrix construction (rho proportional to sum_i_ |psi_i_><psi_i_|), guanine contributes a zero projector at initialization and the final normalization enforces Tr(rho) = 1.

Optional generalized mapping: If one wishes to assign each nucleotide (or local k-mer context) to an arbitrary point on the Bloch sphere, a parameterized encoding can be used: for each base b in {A, C, G, T}, define

$$\left| \psi(b) \right\rangle= \cos\left( \frac{\theta_{b}}{2} \right) \left| 0 \right\rangle+ e^{i \varphi_{b}} \sin\left( \frac{\theta_{b}}{2} \right) \left| 1 \right\rangle$$

where (theta_b_, phi_b_) are fixed constants or derived from normalized biochemical descriptors (e.g., stacking energies, hydrogen-bond pattern, dipole moment, oxidation potential).

qu

## Workflow for Entropy Calculation

1. **Genome and Region Extraction**:
   - The genome is extracted from FASTA files, and annotated regions (coding and non-coding) are segmented based on start and end positions.

## Sequence-to-Qubit Conversion:

- - DNA sequences are mapped into arrays of qubit states based on the predefined mapping.

## Density Matrix Construction:

- - For each region, a density matrix is calculated using the qubit states.

## Entropy Calculation:

- - The eigenvalues of the density matrix are computed, and the von Neumann entropy is calculated.

## Comparative Analysis:

- - Entropies of coding and non-coding regions are compared using statistical tests (e.g., T-test).

## Implementation: Code Workflow

The provided Python script implements the above workflow, and its functionality is summarized below:

## Genome and Regions Extraction:

- - The manual_extract_genome_sequence function reads a genome FASTA file.
  - The extract_region_sequences function extracts coding and non-coding regions based on annotations in a CSV file.

## Sequence Processing:

- - sequence_to_qubits maps sequences to qubit arrays.
  - calculate_density_matrix constructs the density matrix from qubits.
  - von_neumann_entropy calculates entropy from the density matrix.

## Statistical and Visualization Tools:

- - Coding and non-coding entropies are statistically compared using ttest_ind.
  - A histogram visualization contrasts entropy distributions for coding and non-coding regions.

## Summary Outputs:

- - Descriptive statistics (mean, standard deviation, min, max) are computed for both region types.
  - Entropy differences are visualized to highlight distinct quantum properties.

## Entanglement Calculation

Entanglement entropy was calculated to measure the degree of quantum correlation between coding and non-coding regions.

## Mathematical Framework

1. **Joint Density Matrix (ρjoint)**: The joint density matrix represents the quantum state of a combined system consisting of coding and non-coding regions. It is constructed using the tensor product of the qubit states of the two regions:

$$\rho_{\text{joint}}=\frac{1}{n_{1}n_{2}}\sum_{i=1}^{n_{1}} \sum_{j=1}^{n_{2}} \left| \psi_{i}\otimes\phi_{j}\rangle\langle\psi_{i}\otimes\phi_{j} \right|$$

n_1_ and n_2_ are the number of qubits in the coding and non-coding regions, respectively. ∣ψ_i_⟩ and ∣ϕ_j_⟩ are the qubit states of the i-th coding nucleotide and j- th non-coding nucleotide.

1. **Partial Trace**: To calculate the reduced density matrix (ρ_coding_) for the coding region, the non-coding subsystem is traced out:

$$\rho_{\text{coding}}=\mathrm{Tr}_{\text{non-coding}}(\rho_{\text{joint}})$$

Alternatively, the coding subsystem can be traced out to calculate the reduced density matrix for the non-coding region.

1. **Entanglement Entropy (S)**: The entanglement entropy is calculated using the eigenvalues (λ_i_) of the reduced density matrix:

$$S=-\sum_{i} \lambda_{i}\log_{2}\left( \lambda_{i} \right)$$

Where λ_i_>0. This entropy quantifies the amount of quantum information shared between the two regions

## Workflow for Entanglement Calculation

1. **DNA to Qubit Mapping**: Each nucleotide is mapped to a qubit state:

**A (Adenine)**: [1, 0] (|0⟩)

**T (Thymine)**: [0, 1] (|1⟩)

**C (Cytosine)**: [1, 1] (superposition state (|0⟩ + |1⟩)/sqrt(2))

**G (Guanine)**: [0, 0] (erasure/vacuum state |vac>

As above, [0, 0] denotes an erasure/vacuum initialization (zero projector contribution) and the joint/reduced density matrices are normalized so that Tr(ρ) = 1.

1. **Joint Density Matrix Construction**: For every pair of coding and non-coding sequences:
   - Compute the tensor product (⊗) of qubits to form joint states.
   - Use these joint states to construct the joint density matrix (ρ_joint_).
2. **Partial Trace**: To obtain the reduced density matrix:
   - Reshape the joint density matrix into a 4-dimensional array.
   - Compute the reduced density matrix via partial trace: ρ_A_ = Tr_B_(ρ_joint_), where subsystem B is traced out

## Entanglement Entropy Calculation:

- - Compute the eigenvalues λ_i_ of ρ_A_ (Hermitian eigendecomposition) and evaluate the von Neumann entropy S(ρ_A_)=−Σ_i_ λ_i_ log(λ_i_). (Implementation uses standard numerical linear algebra routines.)
  - Calculate entropy based on the eigenvalues.

## Comparison Across Regions:

- - Entanglement entropy was calculated for pairs of coding and non-coding regions.
  - Statistical tests and descriptive summaries were used to analyze the results.

## Implementation: Code Workflow

The numerical workflow implements the coupled evolution defined in Eqs. (Sx-Sy) by constructing the time-dependent Hamiltonian

$$H\left( t \right)=H_{0}+H_{\mathrm{couple}}+H_{\mathrm{pert}}\left( t \right),$$

propagating the state using an explicit first-order update, and renormalizing the state at each step to control numerical drift.

I used different numerical integrators depending on the scenario. For time-independent (unperturbed) Hamiltonians (Section D), I precomputed the exact one-step unitary propagator and applied it iteratively:

$$U = exp(-i H \Delta t/\hbar)$$

For time-dependent driven simulations (Sections E and the driven part of F), I used an explicit first-order update

$$\psi(t+\Delta t) = \psi(t) - \frac{i\Delta t}{\hbar} H(t) \psi(t)$$

followed by renormalization ψ ← ψ / ||ψ||₂ after each step to control numerical drift. In the proton-tunneling model with a continuous coordinate, I used a split-operator (Strang) scheme.

**Algorithmic steps**

1. **Boundary coupling (non-coding to coding):** impose coupling only between the last non-coding site and the first coding site (0-based indices in Python).
2. **State initialization:** build $\psi\left( 0 \right)\in\mathbb{C}^{N}$ from the non-coding and coding subsequences using the nucleotide mapping $\alpha\left( \cdot\right)$ (defined in the main text), then normalize.
3. **Time stepping:** for each time step $t_{n}=n \Delta t$,
   - compute the instantaneous drive

$$d\left( t_{n} \right)=\lambda cos(2\pi f\left( t_{n} \right)t_{n}), f\left( t \right)=f_{0}+kt;$$

- - build the perturbation matrix

$$H_{\mathrm{pert}}\left( t_{n} \right)=d\left( t_{n} \right) \Pi_{\mathrm{nc}},$$

- - where $\Pi_{\mathrm{nc}}$ projects onto the non-coding subspace;
  - propagate the state using the explicit first-order scheme

$$\psi_{n+1}=\left[ I-\frac{i \Delta t}{\hbar}H\left( t_{n} \right) \right]\psi_{n};$$

- - renormalize $\psi_{n+1}\leftarrow\psi_{n+1}/\parallel\psi_{n+1}\parallel$.

**Key implementation snippet (Python; included for reproducibility)**

*# --- 0-based indexing in Python ---*
*# last non-coding site: idx = N_nc - 1*
*# first coding site: idx = N_nc*
N = N_nc + N_c

*# (1) Inter-region boundary coupling: H_couple*
H_couple = np.zeros((N, N), dtype=complex)
H_couple[N_nc - 1, N_nc] = g_nc_c
H_couple[N_nc, N_nc - 1] = g_nc_c

*# (2) State initialization ψ(0) = [ψ_nc(0); ψ_c(0)]*
psi = np.zeros(N, dtype=complex)
psi[:N_nc] = initialize_state(non_coding_sequence) *# α-mapping + internal normalization if desired*
psi[N_nc:] = initialize_state(coding_sequence)
psi /= np.linalg.norm(psi)

*# (3) Time stepping: explicit first-order update + renormalization*
**for** n **in** range(num_steps):
 t = n * dt
 f_t = f0 + k * t
 drive = lambda_perturbation * np.cos(2 * np.pi * f_t * t)

 *# Non-coding-only diagonal driving term: H_pert(t) = drive * Π_nc*
 H_pert = np.zeros((N, N), dtype=complex)
 H_pert[:N_nc, :N_nc] = np.eye(N_nc) * drive

 *# Total Hamiltonian at time t*
 H_t = H0 + H_couple + H_pert

 *# Explicit Euler step: ψ(t+dt) ≈ [I - i dt/ħ H(t)] ψ(t)*
 psi = psi - (1j * dt / hbar) * (H_t @ psi)

 *# Renormalize to control numerical drift*
 psi /= np.linalg.norm(psi)

**Note (implementation correctness):** the perturbation term should be recomputed at each time step and assembled into $H\left( t \right)$; it should not be added cumulatively to an already-perturbed Hamiltonian object across iterations (unless the code explicitly resets the Hamiltonian each step). The snippet above uses a fresh $H_{\mathrm{pert}}$ and forms $H_{t}=H_{0}+H_{\mathrm{couple}}+H_{\mathrm{pert}}$ at each $t_{n}$, matching the mathematical definition of $H\left( t \right)$.

## Functions:

- - sequence_to_qubits: Maps DNA sequences to qubit arrays.
  - joint_density_matrix: Constructs the joint density matrix for coding and non-coding qubits.
  - partial_trace: Computes the reduced density matrix by tracing out one subsystem.
  - entanglement_entropy: Calculates entropy from the reduced density matrix.
  - compute_entanglement_for_genome: Processes coding and non-coding region pairs and calculates their entanglement entropy.

## Sample Analysis:

- - Input: DNA sequences for the first five coding and non-coding regions.
  - Output: Entanglement entropy values for each pair.
  - Results: Displayed in a DataFrame for easy comparison.

## Quantum state evolution over time: implicit perturbations

The simulations provide insights into how DNA’s coding and non-coding regions dynamically respond to internal quantum interactions.

Real DNA sequences were 500 bp long, including 250 bp of coding and 250 bp of non-coding regions: genome position **1802–2301 (non-coding/intergenic: 1802-2051; coding: 2052-2301** DNA polymerase III subunit beta; protein_id **NP_214516.1).**

## Simulation Framework

1. **Hamiltonian Construction**:

The Hamiltonian (H) defines the energy interactions within the system and is central to governing the time evolution of quantum states.

**Diagonal Elements**: Represent nucleotide-specific binding energies, randomly sampled from a realistic range (0.01-0.05 eV) based on known molecular interactions1-3.

**Off-Diagonal Elements**: Represent nearest-neighbor coupling between adjacent nucleotides, set at 0.025 eV1-3.

The Hamiltonian is a symmetric, tridiagonal matrix, capturing both the local (diagonal) and interactive (off-diagonal) energy contributions within the sequence.

## Mathematical Representation:

$$H=\left[ \begin{matrix} E_{1} & J & 0 & \cdots& 0 \\ J & E_{2} & J & \cdots& 0 \\ 0 & J & E_{3} & \cdots& 0 \\ \vdots& \vdots& \vdots& \ddots& J \\ 0 & 0 & 0 & J & E_{N} \end{matrix} \right]$$

E_i_ represents the binding energy of the i-th nucleotide.

J=0.025 eV is the coupling strength between adjacent nucleotides.

## Initialization of Quantum States:

In quantum mechanics, the state of a system is represented by a **state vector** (ψ) in a **Hilbert space**, which describes the probabilities of all possible states the system can occupy.

**State Vector (ψ)**: The state vector ψ\psiψ is a mathematical representation of the quantum state of the DNA sequence. For a DNA region of length N (number of nucleotides), the state vector is:

$$\psi=\left[ \begin{matrix} \psi_{1} \\ \psi_{2} \\ \vdots\\ \psi_{N} \end{matrix} \right]$$

N is the total number of nucleotides in the region (coding or non- coding). ψi is the complex amplitude associated with the quantum state of the iii-th nucleotide.

**Quantum State Initialization**: Each nucleotide is assigned an initial complex amplitude (ψ_i_) based on the previously described mapping strategy:

- - **Adenine (A)**: ψ_i_=1+0j, representing a pure quantum state ∣0⟩.
  - **Thymine (T)**: ψ_i_=0+1j, representing a pure quantum state ∣1⟩.
  - **Cytosine (C)**: ψ_i_=(1+1j)/sqrt (2), representing a quantum superposition state (∣0⟩+∣1⟩)/sqrt (2).
  - **Guanine (G)**: ψ_i_=0, representing a collapsed quantum state with no probability of being ∣0⟩ or ∣1⟩.

**Normalization Condition**: to ensure the conservation of probability in the quantum system, the state vector is normalized such that the total probability is 1:

$$\sum_{i=1}^{N} \left| \psi_{i} \right|^{2}=1$$

∣ψ_i_∣ is the magnitude (absolute value) of the complex number ψ_i_, representing the probability amplitude of the iii-th nucleotide. ∣ψ_i_∣2 is the probability of the iii-th nucleotide being in its assigned quantum state.

## Quantum evolution via unitary propagator (matrix exponential)

The time evolution of a quantum system is described by the **time-dependent Schrödinger equation**:

$$i\hbar\frac{\partial\psi\left( t \right)}{\partial t}=H \psi\left( t \right)$$

ψ(t): The state vector at time t, representing the quantum state of the DNA region. H: The Hamiltonian matrix, which defines the energy interactions in the system (described in detail in the Hamiltonian section).

i: The imaginary unit sqrt (-1).

ℏ: The reduced Planck constant, 1.0545718×10−34 J\cdotps (or approximately 6.582119569×10⁻¹⁶ eV\cdotps).

**Time Evolution:** the time evolution of the quantum states is computed iteratively using the unitary operator:

$$\psi\left( t+\Delta t \right)=e^{-iH\Delta t/\hbar} \psi\left( t \right)$$

- e−iHΔt/ℏ: The unitary time evolution operator, which advances the state vector by a time step Δt.
- Δt: The time step, chosen to ensure numerical stability and accuracy.

**Matrix Exponential**: the operator U=e−iHΔt/ℏ is precomputed using the scipy.linalg.expm function for efficiency. This operator captures the combined effect of the Hamiltonian (H) on the quantum states over a single time step.

**Normalization:** to prevent numerical drift and ensure the conservation of probability, the state vector is renormalized at each time step:

$$\psi\left( t \right)\leftarrow\frac{\psi\left( t \right)}{\parallel\psi\left( t \right)\parallel}$$

∥ψ(t)∥: The Euclidean norm (magnitude) of the state vector.

## Parameters Used in the Simulations

**Time Step (Δt)**: Set to 1×10−13 s. This value is two orders of magnitude smaller than the shortest oscillation period in the system, ensuring numerical accuracy and stability.

**Number of Steps (N)**: 100,000 time steps are used, resulting in a total simulation time of 10 ns. This duration is appropriate for observing quantum dynamics at molecular scales.

## Output and Analysis

**Quantum State Output**: the simulation produces the time evolution of the state vector ψ(t) for each nucleotide at each time step.

**Amplitude (∣ψ∣|)**: the magnitude of the state vector components, ∣ψi∣, represents the probability amplitude of each nucleotide.

**Phase (arg(ψ))**: the argument of the state vector components, arg(ψ_i_), represents the phase of each nucleotide's quantum state.

**Statistical Comparisons**: amplitudes and phases are compared between coding and non-coding regions using **T-tests** to determine if the means of the two groups are significantly different, and **Mann-Whitney U tests** to assess differences in the distributions of the two groups.

## Quantum state evolution over time: external perturbations (without wave shift)

Real DNA sequences were 500 bp long, including 250 bp of coding and 250 bp of non-coding regions: genome position **1802–2301 (non-coding/intergenic: 1802-2051; coding: 2052-2301** DNA polymerase III subunit beta; protein_id **NP_214516.1).**

## Hamiltonian Construction

As before (for “Quantum state evolution over time: implicit perturbations”), the

Hamiltonian (H) used in the simulation encapsulates the quantum properties of the DNA regions. Diagonal elements represent the nucleotide-specific binding energies, sampled uniformly from the range 0.01 to 0.05 eV, consistent with energy levels observed in molecular quantum systems. Off-diagonal elements represent nearest- neighbor coupling interactions, set to a fixed value of 0.025 eV based on reasonable assumptions for molecular interactions.

## Time Evolution Operator

As before (for “Quantum state evolution over time: implicit perturbations”), the time evolution of the quantum states is governed by the time-dependent Schrödinger equation.

A first-order approximation is used to update the state vector iteratively:

$$\psi\left( t+\Delta t \right)=\psi\left( t \right)-\frac{i \Delta t}{\hbar} H\left( t \right) \psi\left( t \right)$$

At each step, the state is normalized to ensure conservation of probability.

## Implementation of External Perturbation:

A time-dependent perturbation term was introduced into the Hamiltonian:

$$H_{t}=H+diag(\lambda\cos\left( 2\pi f t \right))$$

λ=1×10−4 eV represents the perturbation amplitude, chosen based on the photon energy of a 34 GHz signal (≈1.4×10−5 eV).

f=34×109 Hz corresponds to the frequency of the cosmic radiation.

t is the time variable in seconds.

## Parameter Selection

The time step (Δt) was set to 1×10−13 seconds, ensuring that it is sufficiently small to capture the oscillatory dynamics of the 34 GHz wave (period of ≈2.94×10−11 seconds).

The number of steps was fixed at 100,000, corresponding to a total simulation time of 10 nanoseconds.

## Split-Operator Method

The split-operator method was not used in the simulations for this section as the first- order approximation directly computed the time evolution operator. This decision prioritized computational efficiency while preserving the integrity of the model at the given time resolution.

## DNA Sequence and Initialization

The DNA sequence was divided into non-coding and coding regions to observe differences in their quantum behaviors.

Each nucleotide was initialized with a complex amplitude based on the specific mapping (same mapping strategy showed in previous sections).

## Simulation Output

Quantum amplitudes (∣ψ∣|) and phases (arg(ψ)) were extracted at each time step for all nucleotides. Statistical comparisons (T-tests and Mann-Whitney U tests) were conducted to analyze differences in these properties between coding and non-coding regions.

## Quantum state evolution over time: external perturbations (with wave shift)

- Real DNA sequences were 200 bp long, including 100 bp of coding and 100 bp of non-coding regions: genome position 1952–2151 **(non-coding/intergenic: 1952-2051; coding: 2052-2151** DNA polymerase III subunit beta; protein_id **NP_214516.1).**
- **Control DNA sequences were 200 bp long from the non-coding region** 1600-1799. The first 100 bp of that segment were artificially named as non-coding and the rest of the segment (100 bp) was treated as the coding part.
- The control construction (for this section and the sections below where control sequences are used) is designed to remove biological annotation-driven structure (e.g., true coding constraints) by using a same-length segment drawn from a non-coding region and then splitting it into two halves labeled ‘non-coding’ and ‘coding’ for analysis. This control therefore, tests whether the reported dynamical readouts require biologically annotated segmentation versus an arbitrary partition of a sequence of comparable length. However, it does not, by itself, guarantee matching of all low-order sequence statistics between real and control (or between coding and non-coding halves), and more stringent composition-/correlation-matched surrogate controls are discussed as a limitation and future direction.

**System Segmentation and Initialization**: the DNA sequence was divided into non- coding and coding regions, with each nucleotide initialized using a specific quantum state mapping (same as before). This mapping allows the quantum state vector to capture the unique properties of each nucleotide.

**Hamiltonian Construction**: separate Hamiltonians were constructed for non-coding (H_nc_) and coding (H_c_) regions. As the before sections, **diagonal elements** represent binding energies of nucleotides, sampled randomly from a uniform range of 0.01–0.05 eV. **Off-Diagonal Elements**, represent nearest-neighbor couplings were set to a fixed value of 0.025 eV, modeling quantum interactions between adjacent nucleotides. A combined Hamiltonian (_Htotal_) was created by merging H_nc_ and H_c_ with an additional coupling term (0.01 eV) between the last nucleotide of the non-coding region and the first nucleotide of the coding region.

**External Perturbations and Wave Shift**: a time-dependent perturbation was applied only to the non-coding region to simulate the interaction with a cosmic signal

$$H_{t}\left[ 0:len_{nc},0:len_{nc} \right]=H_{t}\left[ 0:len_{nc},0:len_{nc} \right]+diag(\lambda_{\text{pert}} \cos\left( 2\pi f t \right)\cdot1)$$

λ_pert_: Perturbation amplitude, amplified by the DNA’s antenna gain (∼1.7dBi).

f_t_=f_0_+k⋅t: Time-dependent frequency with an initial frequency (f_0_=34 GHz) and a shift rate (k=−1×1018 Hz/s) modeling the Doppler effect.The frequency shift over the simulation time captures the wave shift due to the universe's expansion.

**Time Evolution via the Schrödinger Equation**: as in the previous sections, the time- dependent Schrödinger equation governs the quantum state evolution. The state vector (ψ) was updated iteratively using a first-order approximation:

$$\psi\left( t+\Delta t \right)=\psi\left( t \right)-\frac{i \Delta t}{\hbar} H\left( t \right) \psi\left( t \right)$$

States were normalized at each time step to ensure conservation of probability. For the driven wave-shift simulations in the site-basis model, we propagated the state using the explicit first-order update shown in the Implementation: Code Workflow, followed by renormalization at each step to control numerical drift.

**Spectral and Statistical Analysis**: Quantum amplitudes and phases were recorded for both regions across time steps. Fourier transforms (FFT) were applied to detrended phase data to analyze spectral power within the Doppler-shifted frequency range.

Statistical tests (T-tests and Mann-Whitney U tests) assessed differences in amplitudes and phases between regions.

## Parameter Selection Justification

**Time Step (Δt)**: Chosen as 1×10−13 s to resolve the oscillatory dynamics of the 34 GHz wave (period ∼2.94×10−11 s).

**Frequency Shift Rate (k)**: Set to −1×1018 Hz/s to ensure a significant shift over the simulation period for computational feasibility.

**Perturbation Amplitude (λpert)**: Scaled by the DNA’s antenna gain to simulate enhanced interaction with cosmic signals.

## Mutations induction by quantum tunneling

- Real DNA sequences were 200 bp long, including 100 bp of coding and 100 bp of non-coding regions: genome position 1952–2151 **(non-coding/intergenic: 1952-2051; coding: 2052-2151** DNA polymerase III subunit beta; protein_id **NP_214516.1).**
- **Control DNA sequences were 200 bp long from the non-coding region** 1600-1799. The first 100 bp of that segment were artificially named as non-coding and the rest of the segment (100 bp) was treated as the coding part.

## Double-Well Potential Construction

The double-well potential models the hydrogen bonds in DNA base pairs, with minima corresponding to the stable proton positions. The potential is defined as:

$$V\left( x \right)=V_{0}\left[ \left( \frac{x}{a} \right)^{4}-2\left( \frac{x}{a} \right)^{2}+1 \right]$$

V_0_ is the potential barrier height, and a is the characteristic length scale.

The parameters were chosen based on the nucleotide pair from previous evidence4: V_0_=0.065 eV for A-T and 0.108 eV for G-C pairs.

a=1×10−10 m.

## Hamiltonian Construction

The Hamiltonian (H) comprises the kinetic energy operator (T) and potential energy operator (V): H=T+V(x)

The kinetic energy operator was constructed using finite difference approximations:

$$T_{i,j}=\left\{ \begin{matrix} \frac{2\hbar^{2}}{m_{p} dx^{2}}, & \text{if }i=j, \\ -\frac{\hbar^{2}}{m_{p} dx^{2}}, & \text{if }\left| i-j \right|=1, \\ 0, & \text{otherwise} \end{matrix} \right.$$

where m_p_ is the proton mass, ℏ is the reduced Planck constant, and dx is the spatial step size.

## Initialization of the Proton's State

The initial proton wave function was localized in one well (e.g., left well) using a Gaussian distribution:

$$\psi\left( x,t=0 \right)=\frac{1}{\sqrt{\sigma} \sqrt{\pi}} \exp\left[ -\frac{\left( x-x_{0} \right)^{2}}{2\sigma^{2}} \right]$$

where x_0_ = −a, σ=5×10−11 m, and the wave function was normalized.

## Time Evolution of the Wave Function

The proton dynamics were governed by the time-dependent Schrödinger equation (as previously described). For the driven wave-shift simulations in the site-basis model, we propagated the state using the explicit first-order update shown in the Implementation: Code Workflow, followed by renormalization at each step to control numerical drift.

## Incorporation of External Perturbations

A time-dependent perturbation was added to the potential to simulate incoming cosmic signals:

$$V_{\text{ext}}\left( x,t \right)=[\lambda_{\text{pert}}+\xi\left( t \right)] cos(2\pi\left( f_{0}+\Delta f \right) t) \frac{x}{a}$$

λ_pert_=eE_0_a⋅gain represents the perturbation amplitude scaled by the DNA's fractal antenna properties (E_0_=10−6 V/m, gain = 101.7/10).

ξ(t): Gaussian noise added to simulate stochastic resonance.

f_0_=34 GHz and Δf=0.0008 Hz model the Doppler shift of cosmic signals over 3 hours.

## Tunneling Probability Calculation

At each time step, the tunneling probability (P_right_) was computed as the probability of the proton being in the right well:

$$P_{\text{right}}\left( t \right)=\int_{0}^{\infty} \left| \psi\left( x,t \right) \right|^{2} dx$$

## Parameter Selection Justification Spatial and Temporal Resolution

N=512 points ensured sufficient resolution of the wave function.

dx≈1.96×10−12 m was selected to balance accuracy and computational efficiency. dt=1×10−15 s enabled resolution of oscillatory dynamics associated with the external field.

## Perturbation Amplitude

λ_pert_ was derived from cosmic signal properties and DNA's fractal antenna behavior to ensure physical relevance.

## Simulation Length

105 steps (0.1 ns) allowed for the observation of tunneling dynamics while maintaining computational feasibility.

**Appendix A: Study Limitations**

The concept of DNA as a fractal antenna is a motivation to explore how a DNA-like quantum system might respond to a very weak, structured electromagnetic background. In vivo, photons in the 34–160 GHz range are strongly attenuated by atmosphere and tissue, and the local field near DNA is dominated by thermal and anthropogenic radiofrequency noise. Nonetheless, there exists a non-zero cosmic and solar contribution in the 1–10 GHz “radio window”, which can reach the ground and penetrate tissue to some depth; I regard this as a minimal, structured component of the ambient field, not as a dominant energy source. The model therefore, treats the external drive as an abstract, weak perturbation whose temporal structure (a slow Doppler-like chirp) is inspired by cosmological expansion, rather than as a literal flux of unattenuated CMB photons acting directly on hydrogen bonds. Within this conceptual setting, DNA is hypothesised to behave as a quantum system that could, in principle, act as a matched filter for such structured perturbations, biasing proton-tunnelling events and, consequently, mutation timing. This speculative bridge between cosmological and biological time is examined here at the level of a toy quantum model, not as an established in vivo mechanism.

Computational simulations were performed to study the main hypothesis. Due to computational power availability, the analyses were conducted on the Mycobacterium tuberculosis genome, which is relatively small and may not represent the diversity and complexity of larger genomes, particularly those of higher organisms with extensive non-coding regions.

The mapping strategy assigns qubit states to nucleotides using a simplified scheme. While there exists evidence suggesting that the mapping strategy is adequate [1–3], there is no experimental validation supporting this specific mapping, and alternative mappings could yield different results. Thus, the complex electronic structures and interactions of DNA bases are reduced to simple qubit states, potentially omitting critical quantum behaviors. Further studied could provide information on alternative mapping strategies. Beyond this theoretical support [1–3], the specific assignments in the mapping strategy are based on the biochemical and quantum-relevant characteristics of each nucleotide:

• Cytosine (C): [1,1] or |0⟩ + |1⟩ (superposition state): Cytosine is the most chemically dynamic nucleotide, frequently undergoing methylation and deamination. This dual behavior makes it a strong candidate for a superposition state, reflecting its epigenetic and quantum chemical duality. It also acts asymmetrically in G≡C hydrogen bonding, which contributes to a complex quantum vibrational profile.

• Adenine (A): [1,0] or |0⟩: Adenine is structurally more stable, with fewer chemical variations and less pronounced involvement in state-altering modifications. It pairs symmetrically with thymine and is less entropically dynamic, making it appropriate for a defined, lower-energy quantum state.

• Thymine (T): [0,1] or |1⟩: Thymine’s complementarity and pairing with adenine is stable and symmetric. It shares features of low entropic fluctuation and a stable π-cloud, warranting its assignment to the other basis state.

• Guanine (G): [0,0] or null/ground state: Guanine is structurally and chemically complex, often undergoing oxidation and exhibiting strong π-π stacking. Its assignment to the null or ground state reflects its lower participation in active quantum state transitions within the chosen scheme. It plays a stabilizing role in DNA structure, consistent with anchoring or background quantum configuration.

While any simplified qubit mapping requires assumptions, this strategy leverages both biochemical function and physical models from quantum biology. It provides a coherent and reproducible framework aligned with foundational quantum DNA research, while remaining flexible for future refinements based on experimental data.

**Sequence-statistics confound (coding vs non-coding):** Because the initial state is a deterministic function of the nucleotide string, and because the Hamiltonian/readouts are evaluated on that evolving state, the simulation is inherently sensitive to classical statistical properties of the sequence (GC content, codon-related 3-nt periodicity in coding regions, and higher-order correlations). Consequently, a separation between ‘coding’ and ‘non-coding’ trajectories could, in principle, arise even in a purely classical order-sensitive dynamical system. For this reason, the present results should be interpreted as demonstrating sequence-dependent dynamical signatures within a quantum-like evolution model, not as definitive evidence that biological DNA performs quantum computation. A more stringent isolation of which sequence features drive the separation requires additional surrogate controls that preserve selected low-order statistics (e.g., mono-/di-nucleotide–preserving shuffles and codon-phase–preserving surrogates) and/or comparison to classical dynamical baselines.

When calculating entanglement between DNA regions, due to computational limitations, only the first five coding and non-coding regions were analyzed, limiting the scope of the study.

The main hypothesis proposes shifted cosmic wave signals at 34 GHz as the main source of incoming information. Nevertheless, 34 GHz signals are heavily attenuated by Earth's atmosphere, particularly by water vapor and oxygen absorption, and further weakened by biological tissues, making it unlikely for these signals to penetrate into cells without significant loss and interference. The present work establishes a conceptual frame to further explore a more realistic environment. Interestingly, water near DNA and within cells can form "coherence domains" that resonate with external electromagnetic fields. These domains could amplify weak external signals, making DNA potentially sensitive to frequencies as low as 1 GHz. Finally, this work shows evidence suggesting that DNA could act as a quantum circuit influenced by external signals. This idea is based on the reception of cosmic signals to measure time, however, other types of electromagnetic signals (from other sources) could be affecting the quantum behavior of DNA.

This work lacks biological experimental evidence. A simple experiment to study the proposed mechanism would be to grow bacteria or culture cells (coming from the same source and under the same conditions) with and without the influence of external electromagnetic fields (Faraday cage). After growing, DNA could be sequenced and mutations could be studied. If differential mutational rates are observed in relation to external electromagnetic field exposure, it could be evidence supporting the idea of how external perturbations could alter biological function.

By acknowledging and addressing these limitations, future research can aim for more realistic simulations that better reflect the true behavior of DNA and its potential quantum properties, thereby providing more reliable insights into the intersection of quantum physics and biology.

## References

1. Rieper, E., Anders, J. & Vedral, V. Quantum entanglement between the electron clouds of nucleic acids in DNA. Preprint at <http://arxiv.org/abs/1006.4053> (2011).
2. Hubač, I., Švec, M. & Wilson, S. Quantum entanglement and quantum information in biological systems (DNA). 61–84 (2017).
3. Riera Aroche, R., Ortiz García, Y. M., Martínez Arellano, M. A. & Riera Leal, A. DNA as a perfect quantum computer based on the quantum physics principles. *Sci Rep* **14**, 11636 (2024).
4. Slocombe, L., Sacchi, M. & Al-Khalili, J. An open quantum systems approach to proton tunnelling in DNA. *Commun Phys* **5**, 109 (2022)
