# Supplementary material for: DNA as a quantum system in evolution
Source: PLoS One. 2026 Mar 20;21(3):e0344520. doi: 10.1371/journal.pone.0344520 (PMC13004412; doi:10.1371/journal.pone.0344520)
Supplement: S1 Table — (DOCX) [file pone.0344520.s002.docx]

**S1 Table** – Quantum evolution over time with Doppler effect for real 200 bp sequence

| **Experiment** | **Results for real sequence** | | | |
| --- | --- | --- | --- | --- |
|  | **statistics for phase and amplitude** | | | **statistics for power** |
| **1** |  | Test Statistic | P value | Power within expected frequency range: |
|  | T-test Amplitude | 2871491 | 0.004997 | Non-coding region: 223878.8500840409 |
|  | T-test Phase | -3798519 | 0.000225 | Coding region: 42394.61497765216 |
|  | Mann-Whitney U Amplitude | 6283000000 | 0.001037 | Power ratio (Non-coding / Coding): 5.280832251974834 |
|  | Mann-Whitney U Phase | 3679000000 | 0.001761 | T-test on power values within expected frequency range: |
|  |  |  |  | Test Statistic: -3.714318652334295, p-value: 0.004762622694160269 |
| **2** |  | Test Statistic | P value | Power within expected frequency range: |
|  | T-test Amplitude | 2.787555 | 6.367871e-03 | Non-coding region: 220041.04611642464 |
|  | T-test Phase | 7.966647 | 2.183710e-12 | Coding region: 136755.29404533232 |
|  | Mann-Whitney U Amplitude | 7059.000000 | 2.094282e-07 | Power ratio (Non-coding / Coding): 1.609013001306438 |
|  | Mann-Whitney U Phase | 7546.000000 | 1.662340e-10 | T-test on power values within expected frequency range: |
|  |  |  |  | Test Statistic: -5.183087250766196, p-value: 0.0005174622379350972 |
| **3** |  | Test Statistic | P value | Power within expected frequency range: |
|  | T-test Amplitude | -2.799570 | 6.163645e-03 | Non-coding region: 71431.88724301358 |
|  | T-test Phase | 11.643413 | 6.649703e-21 | Coding region: 654277.2041058297 |
|  | Mann-Whitney U Amplitude | 3382.000000 | 1.138900e-04 | Power ratio (Non-coding / Coding): 0.10917679355898732 |
|  | Mann-Whitney U Phase | 8824.000000 | 1.485942e-21 | T-test on power values within expected frequency range: |
|  |  |  |  | Test Statistic: -5.1161544218033415, p-value: 0.0005983273770406996 |
| **4** |  | Test Statistic | P value | Power within expected frequency range: |
|  | T-test Amplitude | 3.158242 | 2.104691e-03 | Non-coding region: 238168.922937889 |
|  | T-test Phase | 9.975802 | 9.414982e-17 | Coding region: 63614.783789739595 |
|  | Mann-Whitney U Amplitude | 5875.000000 | 2.284830e-02 | Power ratio (Non-coding / Coding): 3.7439241124372598 |
|  | Mann-Whitney U Phase | 8802.000000 | 2.500836e-21 | T-test on power values within expected frequency range: |

|  |  | | | Test Statistic: -5.784005050760849, p-value: 0.00024332032615076714 | |
| --- | --- | --- | --- | --- | --- |
| **5** |  | Test Statistic | P value | Power within expected frequency range: |  |
|  | T-test Amplitude | -2.66148 | 9.092401e-03 | Non-coding region: 72007.67485847526 |  |
|  | T-test Phase | 1.723141 | 8.773755e-02 | Coding region: 57605.147523512955 |  |
|  | Mann-Whitney U Amplitude | 1681.000000 | 8.520181e-16 | Power ratio (Non-coding / Coding): 1.2500215337367822 |  |
|  | Mann-Whitney U Phase | 4834.000000 | 7.761488e-01 | T-test on power values within expected frequency range: |  |
|  |  |  |  | Test Statistic: 4.536520866208821, p-value: 0.0013402160416431453 | |
| **6** |  | Test Statistic | P value | Power within expected frequency range: |  |
|  | T-test Amplitude | -3.044177 | 2.995932e-03 | Non-coding region: 45169.63043242791 |  |
|  | T-test Phase | 2.675847 | 8.476972e-03 | Coding region: 80122.43731700505 |  |
|  | Mann-Whitney U Amplitude | 2349.000000 | 1.533663e-10 | Power ratio (Non-coding / Coding): 0.563757568353967 |  |
|  | Mann-Whitney U Phase | 5877.000000 | 2.255523e-02 | T-test on power values within expected frequency range: |  |
|  |  |  |  | Test Statistic: 3.249488870923804, p-value: 0.009450276367621423 |  |
| **7** |  | Test Statistic | P value | Power within expected frequency range: |  |
|  | T-test Amplitude | 3.515522 | 6.637490e-04 | Non-coding region: 221243.21303915256 |  |
|  | T-test Phase | -4.948870 | 2.836116e-06 | Coding region: 180420.353975477 |  |
|  | Mann-Whitney U Amplitude | 7124.000000 | 8.757249e-08 | Power ratio (Non-coding / Coding): 1.2262652642241478 |  |
|  | Mann-Whitney U Phase | 3426.000000 | 1.764135e-04 | T-test on power values within expected frequency range: |  |
|  |  |  |  | Test Statistic: -4.11044209620737, p-value: 0.00262409086917281 |  |
| **8** |  | Test Statistic | P value | Power within expected frequency range: |  |
|  | T-test Amplitude | -3.427905 | 8.907960e-04 | Non-coding region: 262857.4862739673 |  |
|  | T-test Phase | -5.935473 | 3.555186e-08 | Coding region: 36228.04181472789 |  |
|  | Mann-Whitney U Amplitude | 3377.000000 | 1.082887e-04 | Power ratio (Non-coding / Coding): 7.255636051714699 |  |
|  | Mann-Whitney U Phase | 2772.000000 | 8.293019e-08 | T-test on power values within expected frequency range: |  |
|  |  |  |  | Test Statistic: 5.881131399053351, p-value: 0.00023272873526399023 | |
| **9** |  | Test Statistic | P value | Power within expected frequency range: |  |
|  | T-test Amplitude | 3.067411 | 2.784727e-03 | Non-coding region: 121827.66108365629 |  |
|  | T-test Phase | -12.057665 | 3.246963e-21 | Coding region: 127132.11246072361 |  |
|  | Mann-Whitney U Amplitude | 5736.000000 | 5.314174e-02 | Power ratio (Non-coding / Coding): 0.9582760698741155 |  |

|  | Mann-Whitney U Phase | 656.000000 | 4.110801e-26 | T-test on power values within expected frequency range: |
| --- | --- | --- | --- | --- |
|  |  |  |  | Test Statistic: -5.101342363999706, p-value: 0.0006420102336694913 |
| **10** |  | Test Statistic | P value | Power within expected frequency range: |
|  | T-test Amplitude | -3.134227 | 2.273613e-03 | Non-coding region: 325622.47441018134 |
|  | T-test Phase | 6.850967 | 3.542288e-10 | Coding region: 56981.567196695505 |
|  | Mann-Whitney U Amplitude | 4404.000000 | 1.792952e-01 | Power ratio (Non-coding / Coding): 5.714522966456158 |
|  | Mann-Whitney U Phase | 7148.000000 | 6.307015e-08 | T-test on power values within expected frequency range: |
|  |  |  |  | Test Statistic: 4.370347216037893, p-value: 0.0016970940129112507 |
