# Supplementary material for: DNA as a quantum system in evolution
Source: PLoS One. 2026 Mar 20;21(3):e0344520. doi: 10.1371/journal.pone.0344520 (PMC13004412; doi:10.1371/journal.pone.0344520)
Supplement: S2 Table — (DOCX) [file pone.0344520.s003.docx]

**S2 Table** – Quantum evolution over time with Doppler effect for control 200 bp sequence

| **Experiment** | **Results for control sequence** | | | |
| --- | --- | --- | --- | --- |
|  | **statistics for phase and amplitud** | | | **statistics for power** |
|  |  | Test Statistic | P value | Power within expected frequency range: |
| **1** | T-test Amplitude | -2.571102 | 1.164079e-02 | Non-coding region: 47732.496966155 |
|  | T-test Phase | -0.262759 | 7.932396e-01 | Coding region: 505679.96903908503 |
|  | Mann-Whitney U Amplitude | 2256.000000 | 3.335589e-11 | Power ratio (Non-coding / Coding): 0.09439269871982146 |
|  | Mann-Whitney U Phase | 3989.000000 | 1.805016e-02 | T-test on power values within expected frequency range: |
|  |  |  |  | Test Statistic: -3.835780991927265, p-value: 0.0034851240876469453 |
| **2** |  | Test Statistic | P value | Power within expected frequency range: |
|  | T-test Amplitude | 3.062882 | 2.823462e-03 | Non-coding region: 139149.7334624991 |
|  | T-test Phase | -9.325294 | 6.260113e-16 | Coding region: 48855.08105454799 |
|  | Mann-Whitney U Amplitude | 7991.000 | 7.144176e-14 | Power ratio (Non-coding / Coding): 2.8482141562130403 |
|  | Mann-Whitney U Phase | 1657.000000 | 5.249679e-16 | T-test on power values within expected frequency range: |
|  |  |  |  | Test Statistic: -4.688359236929593, p-value: 0.0008365681860182644 |

| **3** |  | Test Statistic | P value | Power within expected frequency range: |
| --- | --- | --- | --- | --- |
|  | T-test Amplitude | -3.130264 | 2.301651e-03 | Non-coding region: 82061.40663515733 |
|  | T-test Phase | -8.924337 | 6.862709e-15 | Coding region: 212114.5065927697 |
|  | Mann-Whitney U Amplitude | 3961.000000 | 1.495286e-02 | Power ratio (Non-coding / Coding): 0.38687314674193307 |
|  | Mann-Whitney U Phase | 1887.0000 | 4.723059e-14 | T-test on power values within expected frequency range: |
|  |  |  |  | Test Statistic: 3.4513412291911636, p-value: 0.004811409673716123 |
| **4** |  | Test Statistic | P value | Power within expected frequency range: |
|  | T-test Amplitude | 3.534366 | 6.231349e-04 | Non-coding region: 177352.19004071545 |
|  | T-test Phase | 9.393909 | 1.890650e-15 | Coding region: 115379.57265974 |
|  | Mann-Whitney U Amplitude | 6835.000000 | 3.495603e-06 | Power ratio (Non-coding / Coding): 1.537119491365562 |
|  | Mann-Whitney U Phase | 7796.000000 | 2.467447e-12 | T-test on power values within expected frequency range: |
|  |  |  |  | Test Statistic: -4.498839861984879, p-value: 0.0014317019845366049 |
| **5** |  | Test Statistic | P value | Power within expected frequency range: |
|  | T-test Amplitude | 2.727537 | 7.549772e-03 | Non-coding region: 232735.8246072592 |
|  | T-test Phase | 1.576022 | 1.180397e-01 | Coding region: 62307.920176794745 |
|  | Mann-Whitney U Amplitude | 7125.000000 | 8.638887e-08 | Power ratio (Non-coding / Coding): 3.7352526604464753 |
|  | Mann-Whitney U Phase | 4945.000000 | 9.911610e-01 | T-test on power values within expected frequency range: |
|  |  |  |  | Test Statistic: -3.6677318145838633, p-value: 0.004679106358779339 |
| **6** |  | Test Statistic | P value | Power within expected frequency range: |
|  | T-test Amplitude | -3.136275 | 0.002259 | Non-coding region: 891654.928640504 |
|  | T-test Phase | 3.243579 | 0.001584 | Coding region: 2286162.304663469 |
|  | Mann-Whitney U Amplitude | 4248.000000 | 0.084172 | Power ratio (Non-coding / Coding): 0.3900225836204393 |
|  | Mann-Whitney U Phase | 6240.000000 | 0.001501 | T-test on power values within expected frequency range: |
|  |  |  |  | Test Statistic: -3.7810377725313407, p-value: 0.004337721360582997 |
| **7** |  | Test Statistic | P value | Power within expected frequency range: |
|  | T-test Amplitude | 2.819787 | 5.805362e-03 | Non-coding region: 205198.36374317686 |
|  | T-test Phase | 0.463610 | 6.438830e-01 | Coding region: 236787.70369569337 |
|  | Mann-Whitney U Amplitude | 7091.000000 | 1.367611e-07 | Power ratio (Non-coding / Coding): 0.866592143681948 |
|  | Mann-Whitney U Phase | 4818.000000 | 7.461420e-01 | T-test on power values within expected frequency range: |

|  | Test Statistic: -5.3582576128834685, p-value: 0.0003293182367008677 | | | | |
| --- | --- | --- | --- | --- | --- |
| **8** |  | Test Statistic | P value | Power within expected frequency range: |  |
|  | T-test Amplitude | 3.612074 | 4.791619e-04 | Non-coding region: 22178340.53582459 |  |
|  | T-test Phase | -7.784024 | 6.882180e-12 | Coding region: 20409410.86517613 |  |
|  | Mann-Whitney U Amplitude | 5399.000000 | 2.695365e-01 | Power ratio (Non-coding / Coding): 1.0866722553793418 |  |
|  | Mann-Whitney U Phase | 2523.000000 | 2.320316e-09 | T-test on power values within expected frequency range: |  |
|  |  |  |  | Test Statistic: 2.6772389803661416, p-value: 0.025271976849958293 |  |
| **9** |  | Test Statistic | P value | Power within expected frequency range: |  |
|  | T-test Amplitude | -2.999021 | 3.433341e-03 | Non-coding region: 1284720.0874075072 |  |
|  | T-test Phase | -3.972910 | 1.201006e-04 | Coding region: 1201749.3655632348 |  |
|  | Mann-Whitney U Amplitude | 3420.000000 | 1.663035e-04 | Power ratio (Non-coding / Coding): 1.0690416190112868 |  |
|  | Mann-Whitney U Phase | 2424.000000 | 5.056172e-10 | T-test on power values within expected frequency range: |  |
|  |  |  |  | Test Statistic: 1.4135694217894552, p-value: 0.1765155357315506 |  |
| **10** |  | Test Statistic | P value | Power within expected frequency range: |  |
|  | T-test Amplitude | 3.203582 | 1.826396e-03 | Non-coding region: 291750.84456815093 |  |
|  | T-test Phase | 13.078652 | 7.220081e-27 | Coding region: 246788.70983024407 |  |
|  | Mann-Whitney U Amplitude | 7441.000000 | 8.722571e-10 | Power ratio (Non-coding / Coding): 1.1821887831450413 |  |
|  | Mann-Whitney U Phase | 9208.000000 | 1.053527e-25 | T-test on power values within expected frequency range: |  |
|  |  |  |  | Test Statistic: -3.549718867511303, p-value: 0.006212635773061272 |  |
