# Supplementary material for: DNA as a quantum system in evolution
Source: PLoS One. 2026 Mar 20;21(3):e0344520. doi: 10.1371/journal.pone.0344520 (PMC13004412; doi:10.1371/journal.pone.0344520)
Supplement: S3 Table — (DOCX) [file pone.0344520.s004.docx]

**S3 Table** – Simulations for mutation induction by quantum tunneling

| **Simulation** | **Real (t-statistic)** | | **Real (p-value)** | | **Control (t-statistic)** | | **Control (p-value)** | | | |
| --- | --- | --- | --- | --- | --- | --- | --- | --- | --- | --- |
| 1 | | -1,46 | | 0,14 | | -4,96 | | 0,000000675 | |  |
| 2 | | 2,15 | | 0,03 | | -4,53 | | 0,00000576 | |  |
| 3 | | 4,021 | | 0,0000579 | | -0,2 | | 0,84 | |  |
| 4 | | 3,73 | | 0,00018 | | 1,93 | | 0,053 | |  |
| 5 | | 2,78 | | 0,005 | | 0,44 | | 0,65 | |  |
| 6 | | 2,801 | | 0,005 | | 0,36 | | 0,71 | |  |
| 7 | | -1,44 | | 0,147 | | -1,72 | | 0,08 | |  |
| 8 | | -0,71 | | 0,47 | | 0,84 | | 0,39 | |  |
| 9 | | 5,47 | | 4,29E-08 | | 0,56 | | 0,56 | |  |
| 10 | | 4,33 | | 0,0000145 | | 0,85 | | 0,39 | |  |
| **Statistical Analysis** | | **Real Sequences** | | **Control Sequences** | | **Paired t-Test on Differences** | | |  |  |
| Mean t-value | | 2,1672 | | -0,543 | | 2,8102 | | |  |  |
| Standard deviation | | 2,509 | | 2,355 | | 2,335 | | |  |  |
| t-statistic | | - | | - | | 3,805 | | |  |  |
| Degrees of freedom | | - | | - | | 9 | | |  |  |
| p-value | | - | | - | | 0,004 | | |  |  |
