# Supplementary material for: DNA as a quantum system in evolution
Source: PLoS One. 2026 Mar 20;21(3):e0344520. doi: 10.1371/journal.pone.0344520 (PMC13004412; doi:10.1371/journal.pone.0344520)
Supplement: S4 Table — (DOCX) [file pone.0344520.s005.docx]

| p-value |  |  | 0,0394 |  |
| --- | --- | --- | --- | --- |

**S4 Table** – Simulations for mutation induction by quantum tunneling in human CRY1 gene **(Assembly: GRCh38;** <https://www.ncbi.nlm.nih.gov/gene/1407>**)**

- Real sequence (200 bp): **Noncoding (5′ UTR):** chr12: **107,093,061–107,092,962** (100 bp)**. Coding (CDS; starts at ATG):** chr12: **107,092,961–107,092,862** (100 bp)
- Control sequence (200 bp non-coding segment): chr12:107,093,549–107,093,350 (minus strand). The first 100 bp were artificially named as non-coding and the rest of the segment was treated as the coding part.

| **Simulation** | | | **Real (t-statistic)** | | | | **Real (p-value)** | | | | **Control (t-statistic)** | | | | **Control (p-value)** | | | | | | | |
| --- | --- | --- | --- | --- | --- | --- | --- | --- | --- | --- | --- | --- | --- | --- | --- | --- | --- | --- | --- | --- | --- | --- |
| 1 | | | | 4,09 | | | | 0,002 | | | | 0,61 | | | | 0,92 | | | |  |  |  |
| 2 | | | | 1,99 | | | | 0,00041 | | | | 0,79 | | | | 0,81 | | | |  |  |  |
| 3 | | | | -0,91 | | | | 0,32 | | | | -2,72 | | | | 0,16 | | | |  |  |  |
| 4 | | | | 3,02 | | | | 0,0007 | | | | 0,39 | | | | 0,59 | | | |  |  |  |
| 5 | | | | 6,28 | | | | 0,00063 | | | | -4,81 | | | | 0,0000286 | | | | |  |  |
| 6 | | | | -1,22 | | | | 0,11 | | | | 0,96 | | | | 0,78 | | | |  |  |  |
| 7 | | | | 5,58 | | | | 0,0000837 | | | | -0,62 | | | | 0,95 | | | |  |  |  |
| 8 | | | | 5,64 | | | | 0,00000467 | | | | 0,85 | | | | 0,43 | | | |  |  |  |
| 9 | | | | 2,68 | | | | 0,000067 | | | | -5,96 | | | | 0,000075 | | | |  |  |  |
| 10 | | | | -0,64 | | | | 0,593 | | | | 2,78 | | | | 0,089 | | | |  |  |  |
| **Statistical Analysis** | | | | **Real Sequences** | | | | **Control Sequences** | | | | **Paired t-Test on Differences** | | | | | | |  |  |  |  |
| Mean t-value | | | | 2,651 | | | | -0.773 | | | | 3,424 | | | | | | |  |  |  |  |
| Standard deviation | | | | 2,835 | | | | 2.808 | | | | 4,497 | | | | | | |  |  |  |  |
| t-statistic | | | |  | | | |  | | | | 2,408 | | | | | | |  |  |  |  |
| Degrees of freedom | | | |  | | | |  | | | | 9 | | | | | | |  |  |  |  |
